# Interactome of SARS-CoV-2 / nCoV19 modulated host proteins with computationally predicted PPIs

**DOI:** 10.21203/rs.3.rs-28592/v1

**Published:** 2020-05-13

**Authors:** Kalyani B. Karunakaran, N. Balakrishnan, Madhavi K. Ganapathiraju

**Affiliations:** 1 Supercomputer Education and Research Centre, Indian Institute of Science, Bangalore, 560 012, India; 2 Department of Biomedical Informatics, School of Medicine, University of Pittsburgh, Pittsburgh, USA; 3 Intelligent Systems Program, School of Computing and Information, University of Pittsburgh, Pittsburgh, USA

## Abstract

World over, people are looking for solutions to tackle the pandemic coronavirus disease (COVID-19) caused by the virus SARS-CoV-2/nCoV-19. Notable contributions in biomedical field have been characterizing viral genomes, host transcriptomes and proteomes, repurposable drugs and vaccines. In one such study, 332 human proteins targeted by nCoV19 were identified. We expanded this set of host proteins by constructing their protein interactome, including in it not only the known protein-protein interactions (PPIs) but also novel, hitherto unknown PPIs predicted with our High-precision Protein-Protein Interaction Prediction (HiPPIP) model that was shown to be highly accurate. In fact, one of the earliest discoveries made possible by HiPPIP is related to activation of immunity upon viral infection. We found that several interactors of the host proteins are differentially expressed upon viral infection, are related to highly relevant pathways, and that the novel interaction of NUP98 with CHMP5 may activate an antiviral mechanism leading to disruption of viral budding. We are making the interactions available as downloadable files to facilitate future systems biology studies and also on a web-server at http://hagrid.dbmi.pitt.edu/corona that allows not only keyword search but also queries such as “PPIs where one protein is associated with ‘virus’ and the interactors with ‘pulmonary’”.

## Introduction

COVID-19 (Coronavirus Disease 2019) is an infectious virus outbreak which emerged as an epidemic in one city in December 2019 and within 3 months swept across 213 countries and territories into a pandemic global health crisis with more than 3 million confirmed infected cases and 217,000 deaths as of April 30, 2020 (WHO). The novel coronavirus (SARS-CoV-2/nCoV-19) has been identified as the causative agent of this coronavirus disease named COVID-19 (19 refers to the year 2019 when it started).^1^ nCoV19 belongs to the large family of coronaviruses (coronaviridae) known to be pathogenic in mammals and birds. They are enveloped viruses with a positive-sense single-stranded RNA genome of 26.2–31.7 kilobases.^2^ In humans, they cause respiratory infections ranging from the common cold to possibly-fatal acute respiratory distress syndrome (ARDS) and acute lung injury (ALI), which are noted in COVID-19 as well as in its predecessors, namely, SARS (severe acute respiratory syndrome, 2002–2003) and MERS (middle east respiratory syndrome, 2012).^3^ nCoV19 is airborne, and causes no symptoms in several infected people who may become silent carriers of the disease to more vulnerable population; COVID-19 is spreading at an exponential rate globally, prompting efforts to develop technologies, therapeutics and vaccines to prevent it from reaching its projected peak. Scientists across the globe are studying the genetic features of nCoV19 and investigating the mechanisms of its host invasion and host-response to viral infection, in hopes of discovering treatment strategies to combat the outbreak.

The viral infection sets off a cascade of interactions among multiple genes and proteins in the host cell. This complex network has the potential to restrict viral replication in host cells, or conversely to be taken over by the virus for its replication. Several research groups have studied the effects of nCoV19 on the host from a systems-level perspective.^4–6^ 332 human proteins that bind to nCoV19 proteins were identified through affinity purification – mass spectrometry (AP-MS) by Gordon et al.^6^ Melo et al. identified 120 genes differentially expressed in the A549 human cell line on nCoV19 infection.^5^ Bojkova et al. monitored nCoV19 infection in Caco2 cell line and generated temporal infection profiles of 2,687 genes in the host translatome and 6,258 gene in the proteome.^7^ Data generated by these studies can be employed to conduct a systematic, unbiased and data-driven investigation into COVD-19 from the perspective of the host, by constructing the relevant protein interactome (i.e. protein-protein interaction network).

Protein-protein interactions (PPIs) drive the cellular machinery and facilitate biological processes including signal transduction, formation of cellular structures and enzymatic complexes. When viral proteins bind to some proteins in the host cell, this effect spreads through regulatory and biophysical interactions along the interactome affecting other proteins in the PPI network, posing deeper implications for the infection, host immunity, and the effect of therapeutics.^8^ Despite being critical to unravelling novel disease mechanisms and drugs, ~85% of estimated PPIs are currently unknown and several disease-associated genes have no known PPIs. More than ~600,000 PPIs are said to exist in the human interactome^9^ and only ~90,000 PPIs are known from PPI repositories such as HPRD^10^ and BioGRID^11^. Detecting the PPIs using experimental techniques such as co-immunoprecipitation (Co-IP)^12,13^ are prohibitively laborious and time-consuming at large scale. Tens of thousands of PPIs are being added into the interactome through systematic high throughput studies with yeast two hybrid system^14^ and AP–MS^15^. A large part of the interactome (>70–80%) remains unknown yet. Hence, computational algorithms have been developed to predict PPIs in human as well as model organisms.^16–24^ We developed a computational model called **HiPPIP** (high-precision protein-protein interaction prediction) that was deemed highly accurate by computational evaluations, and experimental validations of some pairs (sixteen PPIs were tested by experiments and all sixteen were shown to be true PPIs (Supplementary Table S1)).^25,26^ HiPPIP computes features of protein pairs such as cellular localization, molecular function, biological process membership, genomic location of the gene, and gene expression in microarray experiments, and classifies the pairwise features as *interacting* or *non-interacting* based on a random forest model.^25^ The threshold of HiPPIP to classify a protein-pair as “a PPI” was set high in such a way that it yields very high-precision even if low recall. Some of the PPIs predicted by HiPPIP proved to be of high translational impact. For example, we predicted that the human OASL protein (*IFN-inducible oligoadenylate synthetases-like)* interacts with RIG-I (*retinoic acid-inducible gene I*); it was validated to be a true PPI. Further investigations conclusively showed that this interaction is responsible for activating cellular innate immunity to virus infections:^26^ OASL enhances antiviral signalling mediated by the viral RNA sensor RIG-I by binding through its C-terminal ubiquitin-like domain.^26^ Other high-impact results from interactome analysis include shared PPIs explaining inverse epidemiological relationship between schizophrenia and rheumatoid arthritis^27^ and cilia-transduced cell signaling in congenital heart disease,^28,29^ and more.^30^

**Figure 1.**
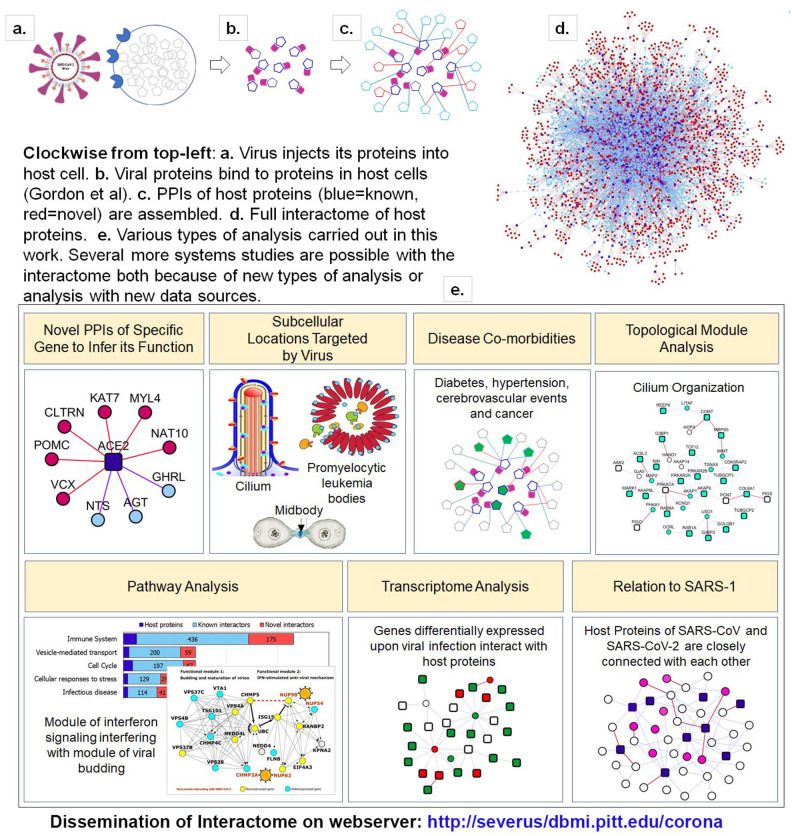
Concept diagram of the analysis presented in the paper.

In this work, we present the host protein interactome (HoP Interactome) of nCoV19, namely PPIs of the 332 human proteins that the virus proteins bind to (Gordon et al.^6^). Key contributions of this work are a set of novel PPIs that are computationally predicted with high-precision, combined with known PPIs from public databases, and the results of analyzing the network of interactions with functional annotations and with nCoV19-relevant transcriptomic and proteomic data. Importantly, we are making this interactome, including about two thousand hitherto unknown PPIs with rich annotations on a webserver with search capabilities to the scientific community and in graph formats amenable for computational analyses. A concept diagram of analysis carried out here is shown in Figure 1.

At a time when biomedical science researchers have closed their labs to practice social distancing,^31^ this digital resource presents new computationally derived information at their fingertips, presenting an avenue for continued scientific enquiry. A biomedical scientist typically has undeterred focus, say on a specific protein or a pathway even when broadly interested in the study of a specific disease. When presented with numerous computational results about the protein or pathway, the biomedical scientist typically needs to formulate a functional hypothesis about what the prediction entails if/after validated to be true. That is, the experiment is built around a hypothesis formulated by the scientist, for which one of the computational results serves as the seed. We believe that it would be very timely and of immense value to present these computational results to the biomedical community, when the scientists are currently having to work in a digital mode away from their wet labs. Many of these two thousand novel PPIs may serve as seeds for new directions to science, similar to how OASL-DDX58 interaction has advanced the study of the role of OASL proteins in viral immunity.^26^

## Results

**Figure 2.**
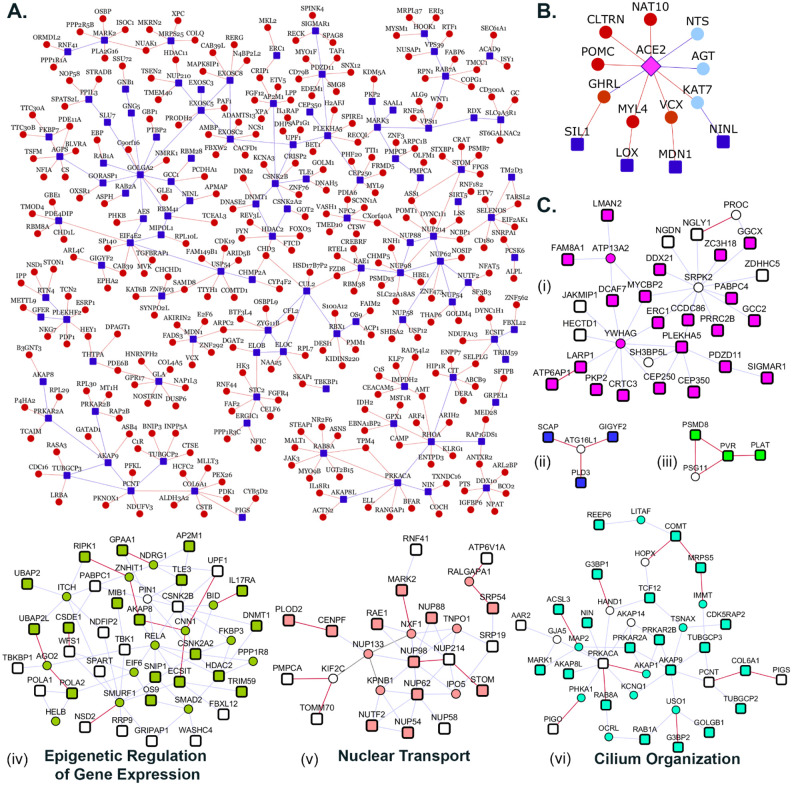
Network views of protein-protein interactions: **A**. **Partial view of the HoP interactome:** Genes are shown as nodes and PPIs as edges. As the full network is very large, only a partial view showing a large connected component of novel interactors and their neighbors, all of which have transcriptomic/proteomic evidence related to nCov19 (Supplementary Data File S2), are shown. Dark blue square-shaped nodes: host proteins; red nodes/edges: novel interactors/interactions; blue nodes/edges: known interactors/interactions. **B**. **ACE2 Interactome**: PPIs of ACE2 protein, extended to show four host proteins that are two-edges away from it. Color legend is as in A. **C**. **Modules identified from network topology**: Six out of seventeen total modules each with 3 or more nodes are shown, whereas remaining modules are shown in Supplementary Figure S2. Each module is depicted in a different color. Within each module, colored nodes depict genes with at least one evidence of transcriptomic/proteomic relevance to nCoV19.

We collected 332 host proteins that were identified to interact with 27 nCoV19 viral proteins by Gordon et al.^6^ To assemble the interactome of these host proteins, we compiled known PPIs from HPRD^10^ (Human Protein Reference Database) and BioGRID^11^ (Biological General Repository for Interaction Datasets) and predicted novel PPIs by applying the HiPPIP algorithm described earlier.^32^ Note that the interactome is human protein interactome, and not a host-virus interactome; the relevance to COVID19 is that the core proteins for which the interactome is assembled are those that viral proteins bind to. HiPPIP predicted ~2,600 PPIs of which ~600 PPIs are previously cataloged in HPRD and BioGRID, leaving ~2,000 PPIs to be considered as novel PPIs of the host proteins. There are an additional 3,500 PPIs that are known and not predicted by HiPPIP (this is as it should be: HiPPIP prediction threshold has already been fixed^32^ to achieve *high precision* by compromising *recall*, which is required for adoption into biology; in other words, it is set to predict only a few PPIs out of the hundreds of thousands of unknown PPIs, but those will be highly accurate). As reported in Supplementary File 1, prior validations of 16 PPIs predicted by HiPPIP in our other studies validated all 16 to be true; the experiments were carried out by diverse research labs. Overall, the host protein (HoP) interactome consists of 4,408 proteins and 6,076 interactions (Supplementary Data File 1). A partial network of host proteins and their novel interactors is shown in Figure 2A (see Supplementary Figure S1 for the full network of novel interactors).

We verified whether any of the 2,000 novel PPIs came up in recent interactome maps HuRI (HI-Union)^14^ and BioPlex^15^. While there was no overlap with HuRI union dataset, there were 8 PPIs in the BioPlex map (ADAM9-ADAM32, P3H3-OS9, PVR-NECTIN2, SRRM2-SNIP1, PABPC4-LUC7L2, PRKACA-AKAP1, NDUFA13-ECSIT, and NPTX1-NPTX2). The small overlap is not surprising because even high-throughput biotechnological methods discover different parts of the interactome with only small overlaps with each other6, this demonstrating complementary strengths.^14^

Applications of this network are two-fold: (1) biologists, who typically focus their research on specific proteins or a pathway may look up the novel interactions relevant to that protein or pathway (e.g.^26^) (2) computational systems biologists may investigate it in conjunction with transcriptomic/proteomic data (e.g.).^30,33–35^ To facilitate ‘(1)’, we are making these results available over an interactive webserver, and to facilitate ‘(2)’, we are releasing the data as downloadable files in various formats.

We employed ‘Netbox’^36^ to identify modules based on network topology. It expands the core proteins by adding nodes from the interactome whose number of links to core proteins are statistically significant compared to its degree in the human interactome. From this network, it identifies highly interconnected modules. It was able to connect 323 proteins (220 host proteins and 103 linker proteins) into 21 modules, of which 14 modules had 4 or more nodes each (Supplementary Figure S2). For comparison, when novel PPIs are not included, it connects 199 proteins (138 host proteins and 61 linker proteins) into 18 modules of which 10 had 4 or more proteins each. Scaled modularity score (z-score compared to random networks) was 17.0 with novel PPIs, and it was 14.5 without novel PPIs (z-score compared to corresponding random networks). Bioinformatic analysis of the computed modules showed that five modules formed with novel interactors had statistically significant enrichment of Gene Ontology biological process terms: *epigenetic regulation of gene expression* (p-value=3.3E-04, odds ratio=10.4), *nuclear transport* (p-value=2.4E-12, odds ratio=21.6), *cilium organization* (p-value=1.28E-03, odds ratio= 7.8) *ribonucleoprotein complex biogenesis* (p-value=0, odds ratio=22.4), and *vesicle-mediated transport between endosomal compartments* (p-value=9.4E-06, odds ratio=123.4) (Figure 2C i-vi). When novel PPIs are excluded, some of these associations were missed and the modules were smaller, but three additional functional modules were found: *cell cycle G2/M phase transition* (p-value=0.0019, odds ratio=21.7, 20 proteins), *DNA replication* (p-value=0.0049, odds ratio=55.25, 3 proteins) and *cell-cell signaling by Wnt* (p-value=0.0049, odds ratio=9.3, 24 proteins) (Supplementary Table S2).

### ACE2 Interactome

SARS-CoV-2 engages the host receptor ACE2 (*angiotensin-converting enzyme 2*) for cell entry.^37^ Viral entry happens prior to the interaction of the viral proteins with host cellular proteins; it was the latter that was studied by Gordon et al.^6^ Therefore, it was not part of the 332 core genes considered in constructing the interactome. Owing to its crucial role in nCov19 infection, we assembled its known and novel PPIs separately and found that it was connected to four host proteins (SIL1, LOX, MDN1 and NINL) through an intermediate interactor, i.e. separated by two edges, where one or both intermediary PPIs are novel predicted ones (see red edges in Figure 2B).

These connections reveal interesting insights: ACE2 is a key player of the renin-angiotensin hormone system that regulates blood pressure and electrolyte balance.^38^ In line with this, we found that its interactors AGT (*angiotensin*), GHRL, CLTRN and POMC are associated with the Reactome Pathway *peptide hormone metabolism* (p-value=2.9E-05). ACE2 and its interactors were also enriched in the Gene Ontology Biological Process *circulatory system process* (ACE2, AGT, NTS, POMC, GHRL and the host protein MYL4; p-value=0.001). Three host proteins are associated with numerous vascular and cardiac phenotypes: LOX with *abnormality of blood volume homeostasis*, *aortic root aneurysm*, *ascending aortic dissection*, *carotid artery dilatation*, *coronary artery atherosclerosis*, *cystic medial necrosis of the aorta*, *descending thoracic aorta aneurysm*, *dilatation of the cerebral artery*, *left ventricular failure*, *peripheral arterial stenosis*, MYL4 with *paroxysmal atrial fibrillation* and *bradycardia*, and SIL1 with *abnormal aldolase level*.

The co-morbidity of hypertension, diabetes and cardiovascular among the group of COVID19 patients with high fatality rate warrants a closer look at ACE2 and other host proteins linked to cardiac and vascular phenotypes.

### Wiki-CORONA: A web server of novel host PPIs

The HoP interactome is available on a website called *Wiki-CORONA* (http://hagrid.dbmi.pitt.edu/corona/). It has advanced-search capabilities, and presents comprehensive annotations, namely Gene Ontology, diseases, drugs and pathways, of the two proteins of each PPI side-by-side. Here, a user can query for results such as “PPIs where one protein is anti-viral and the other is involved in immunity”, and then see the results with the functional details of the two proteins side-by-side. The PPIs and their annotations also get indexed in major search engines like Google and Bing. Querying by biomedical associations is a unique feature which we developed in *Wiki-Pi* that presents known interactions of all human proteins.^39^

### Transcriptome Analysis

Significantly large number of proteins in the interactome were differentially expressed in epithelial cells infected with SARS coronavirus (GSE17400, Calu-3 cell, 48 hours post-infection; p-value=4.76E-12). Several proteins also showed differential expression in the transcriptome level after infection by Urbani strain of SARS coronavirus (GSE37827, Calu-3 cells, 72 hours post-infection) and in peripheral blood mononuclear cells of SARS patients (GSE1739^40^). These latter two datasets of differential expression did not show statistically significant overlaps; yet, the transcriptomic evidence highlights key protein-encoding genes associated with viral infection that interact with the core proteins considered in this study. As several of the interactors here are revealed through computational prediction, the information that they are differentially expressed in SARS/SARS-Cov-2 infections presents opportunity to prioritize consideration of novel PPIs for further study.

Melo et al. had identified 120 differentially expressed genes (DEGs) associated with nCoV19 infection in the A549 cell line.^5^ Of these, only 2 were common with the 332 host proteins identified through AP-MS study^6^ (‘host proteins’). However, our study revealed several interesting links between the two sets: (a) 31 DEGs are *direct interactors* of 38 host proteins, with some DEGs interacting with multiple host proteins; (b) Thirteen novel PPIs exist between the two sets: AAR2-SAMHD1, TUBGCP2-C1R, IMPDH2-C1S, GOLGA7-TCIM, RAB8A-STEAP1, GDF15-EHF, REEP5-PDK4 FAM162A-PARP14, STOML2-CDH1, FGA-RAB14, FBXL12-C19orf66, ECSIT-C19orf66 and EIF4H-PTPN12. (c) 108 DEGs and 285 host proteins are highly interconnected through 808 common interactors (statistically significant overlap with odds ratio=1.5, p-value=7.12E-54). (d) Pathway enrichment analysis of overlapping interactome (consisting of shared interactors and the DEGs and host proteins that they interact with) revealed several immune-related pathways with FDR-corrected p-value<0.05.

2,630 proteins in the interactome that are supported by the above mentioned transcriptomic and proteomic evidence are listed in Supplementary Data File S2. In fact, the selected novel interactors shown in Figure 2A all have transcriptomic/proteomic evidence.

**Figure 3.**
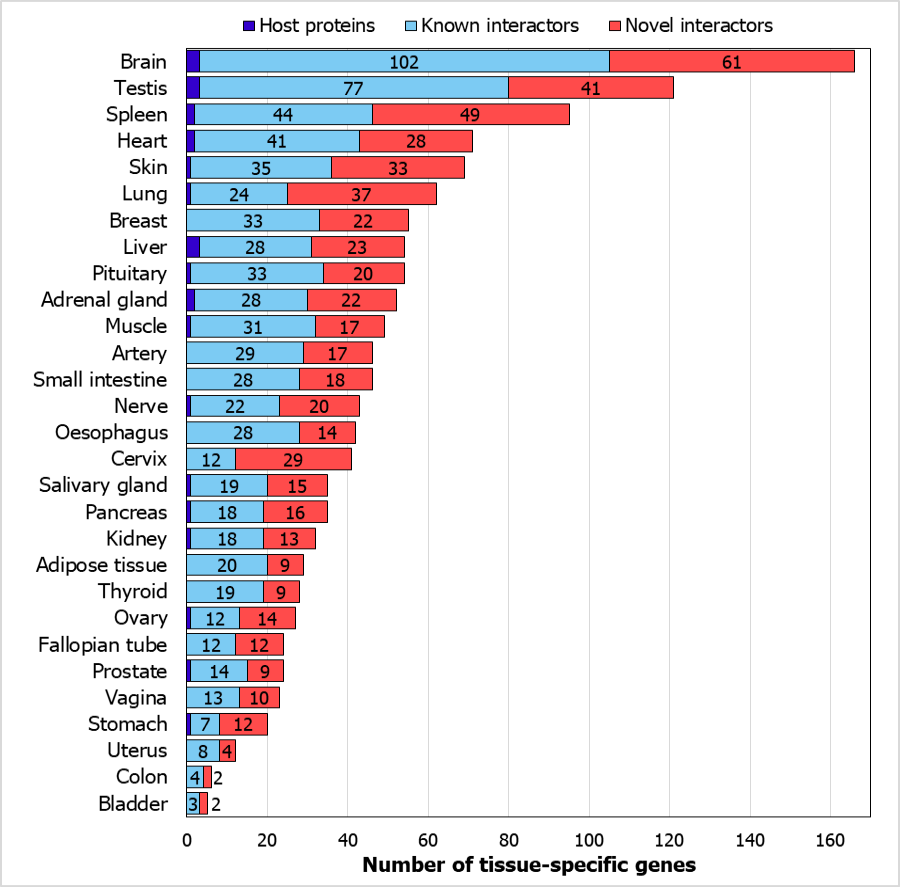
Tissue-specific genes in CoV-HP interactome: Number of genes from the interactome which show tissue specificity are shown. The genes show at least 5-fold

We studied tissue specific expression of the proteins in the interactome using GTEx data.^41^ Genes with an expression level greater than 1 TPM (transcripts per million) and relative expression at least 5-fold higher in a particular tissue (tissue-enriched) or a group of 2–7 tissues (group-enriched) were considered. As expected, many genes showed specific expression in lung which is the target tissue of the virus, and in spleen which regulates the immune response of the host (Figure 3). New PPIs were found of host proteins with 37 lung-specific proteins and 49 spleen-specific proteins. Host proteins also interacted with several brain and heart tissue specific proteins, which is of importance as cerebrovascular diseases and coronary heart diseases are co-morbidities among COVID-19 non-survivors.^42^ (Figure 3).

### Gene Ontology Term Enrichment

#### PML bodies and the midbody may function as subcellular targets of nCoV19

Gene Ontology enrichment analysis of the interactome identified several subcellular locations that may be targeted by nCoV19. Cellular locations included points of virus entry such as the *cell-substrate junction*, *nuclear periphery* and specific sites from where viral proteins may potentiate viral replication, gene expression and modulate the immune response of the host such as the *midbody*, *nuclear chromatin* and *PML body* (each term with p-value<0.0001). PML (promyelocytic leukaemia bodies are nuclear sub-compartments that repress viral replication through entrapment or epigenetic silencing of the viral genomes.^43^ Components of PML bodies activate interferon-stimulated genes and cytokines, and may also be upregulated on induction of interferons.^43^ Therefore, it is conceivable that viruses target PPIs in these structures to circumvent anti-viral defences of the host cell. Sixty-one proteins in the HoP interactome are PML components. These include the host protein AKAP8L, which has been known to promote retroviral gene expression, and 55 known interactors and 5 novel interactors (RNF111, SP140, ELF4, NFE2, CIART) of other host proteins. Our model predicted an interaction of EIF4E2 with SP140, an interferon-inducible PML component; nCoV19 may perturb this PPI. The midbody is a microtubule-rich stricture that connects the daughter cells and marks the site of abscission during cytokinesis. Viruses have been known to recruit certain protein complexes that also localize to the midbody during cytokinesis, to the host cell membrane to promote its scission and thereby the release of viruses.^44^ This co-opting of proteins may explain the enrichment of midbody proteins in the HoP interactome. 83 proteins in the HoP interactome, including 11 host proteins (RHOA, CENPF, CIT, RAB8A, NUP62, SCCPDH, SPART, RDX, ARF6, CNTRL and RALA), 63 known interactors and 9 novel interactors (KIF4A, BIRC5, INCENP, ALKHB4, DNM2, DDX11, ARL2BP, ABRAXAS2 and WIS) localize to the midbody.

#### Cell cycle phase transitions in the host may be modulated by nCoV19

Enriched biological processes in the interactome included *(G1/S and G2/M) mitotic cell cycle phase transitions*, *regulation of vesicle-mediated transport*, *covalent chromatin modification* and *nuclear transport* (p-value<0.0001). The response of the host cell to nCoV19 infection has been shown to be significantly delayed and devoid of several anti-viral mechanisms.^5^ During early stages of the infection, it is possible that the virus induces a G1/S phase transition to surreptitiously synergize the replication of the viral genome with that of the host genome.^45^ In the later stages, it may block the G2/M phase transition to maximise the levels of viral genome.^45^ We found novel (predicted) interactions of host proteins with 34 proteins involved in cell cycle phase transition: ANAPC4, ANAPC7, ARPP19, CCNB3, CDC14B, CDC16, CDC7, CEP164, CETN2, CLSPN, CRLF3, DCTN1, DNM2, DYNC1H1, E2F6, ENSA, FBXL7, GFI1, GML, HYAL1, INHBA, JADE1, NEUROG1, NPAT, ORC2, PPM1D, RAD17, SPDYA, TAOK2, TICRR, TRIAP1, XPC, ZFP36L1, ZNF655.

### Pathway Associations

#### Resveratrol-modulated sub-network of genes involved in the tristetraproline pathway

**Figure 4.**
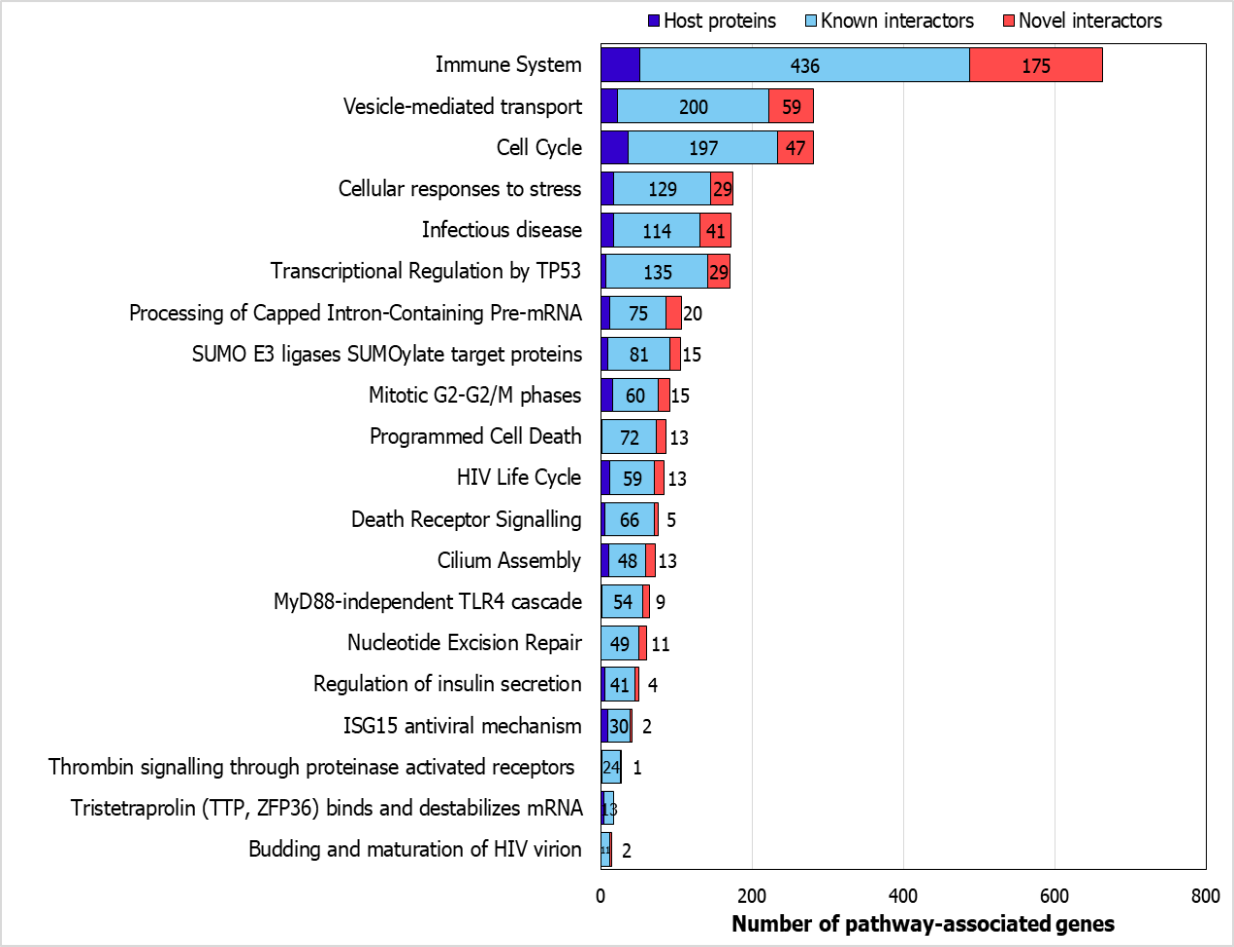
Pathways associated with the CoV-HP interactome; Number of genes from the interactome associated with selected pathways are shown.

Using WebGestalt,^46^ we compiled the list of the Reactome pathways (Figure 4), which showed a statistically significant enrichment of several pathways related to viral entry and infection such as *infectious disease*, *HIV life cycle*, *vesicle-mediated transport* and *membrane trafficking*. Several immunity-related pathways which mediate the host response such as *MyD88 dependent TLR4 signalling* and *ISG15 anti-viral mechanism* were also identified.

**Figure 5.**
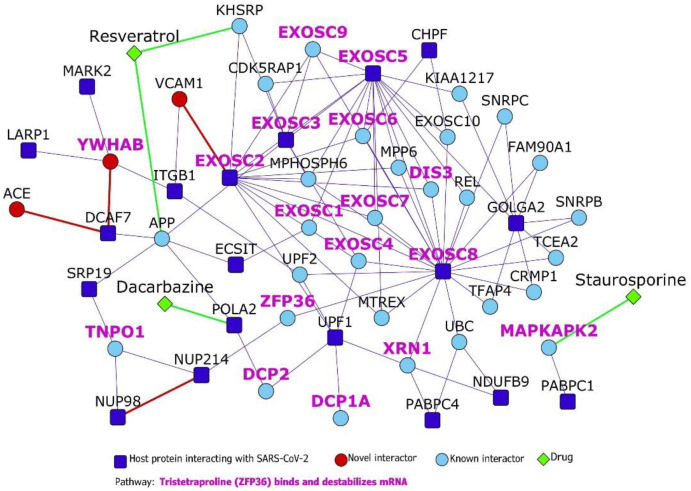
Tristetraproline pathway: Dark blue nodes are host proteins, light-blue nodes are known interactors, and red nodes are novel interactors. Diamond-shaped green nodes depict drugs. Nodes with purple labels are proteins involved in the tristetraproline pathway.

The transcriptional profile of the host cell after nCoV19 infection had revealed a remarkably limited anti-viral response compared to that elicited by seasonal influenza-A and respiratory syncytial viruses.^5^ This prompted us to inspect a posttranscriptional regulatory pathway that was enriched in the HoP interactome, namely, *tristetraproline (ZFP36) binds and destabilizes mRNA* (p-value<0.0001). ZFP36 is an RNA-binding protein that targets AU-rich sites in the mRNA transcripts coding for immune proteins and destabilizes them by promoting the deadenylation of their poly(A) tails.^47,48^ YWHAB increases cytoplasmic localization of ZFP36, possibly preventing destabilization of these genes and attenuation of immune response.^49^ We extracted the direct PPIs of the 17 genes belonging to this pathway from the HoP interactome and isolated this sub-network for further inspection (Figure 5). Our predictions show that the host protein DCAF7, which is known to function as a scaffold protein and a facilitator of PPIs, interacts with YWHAB and ACE1, belonging to the class of receptors targeted by nCoV19 (Figure 5). This raises the possibility that the virus protein Nsp9 (which interacts with DCAF7) may somehow perturb YWHAB-induced cytoplasmic localization of ZFP36 through its action on DCAF7. Nsp9 may activate or promote the sequestration of YWHAB with DCAF7, thereby reducing its capacity to form a complex with YWHAB. YWHAB-mediated destabilization of immune genes may then lead to a weakened immune response, creating a conducive environment for nCoV19 infection. We also identified 3 drugs targeting the proteins in this sub-network using Drug Bank:^50^ resveratrol targeting KHSRP and APP, known interactors of the host protein EXOSC2, which is involved in the tristetraprolin (TTP) pathway, staurosporine targeting TTP-associated MAPKAPK2 which has been predicted to interact with PABPC1 and dacarbazine targeting the host protein POLA2 (Figure 5). Gene expression profiles induced by these drugs in various cell lines were found to have a negative correlation with SARS-associated gene expression profiles, namely, that of lung fibroblast MRC5 cells infected with SARS-CoV and in peripheral blood mononuclear cells of SARS patients (analysis using NextBio; https://www.nextbio.com).^51,52^. Resveratrol has been proposed as a therapeutic option for nCoV19 based on its antagonistic properties against MERS-CoV.^53^

### Genetic Disorder Enrichment Analysis

#### Network proximity of genes associated with diabetes and hypertension to the host proteins

**Figure 6.**
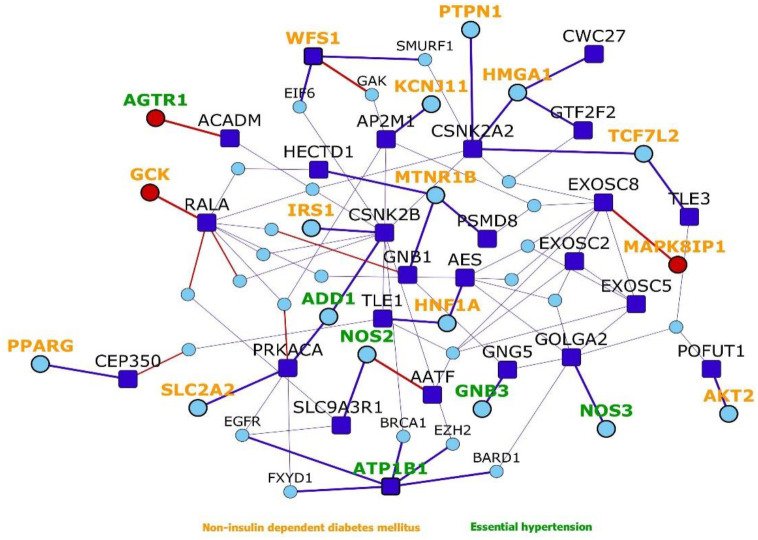
Network proximity of diabetes and hypertension to host proteins: Dark blue nodes are host proteins, light blue nodes are known interactors and red nodes are novel interactors. Nodes with orange labels are diabetes-associated genes, whereas nodes with dark green labels are hypertension-associated genes.

We studied the association of interactome genes with any genetic disorders/traits in the OMIM database. 155 genes in the interactome, including 9 host protein-encoding genes, and 121 known interactors and 25 novel interactors of host proteins, were found to be associated with 35 disorders (overlap of each disease had p-value<0.05). This included 13 types of cancers, 7 metabolic disorders, 4 neurological disorders, 3 developmental disorders, 2 eye-related disorders, 2 vascular diseases, 1 infectious disease, 1 inflammatory disorder, 1 respiratory disorder and 1 skin disease (Figure 6 and Table 1). Some of these diseases enriched in the interactome are co-morbidities among non-survivors and critically ill COVID patients (e.g. diabetes, hypertension, cerebrovascular events and cancer).^42,54^ Thirteen genes in the interactome were associated with *non-insulin dependent diabetes mellitus* (odds ratio=10.8, p-value=4.38E-10), 6 genes with *essential hypertension* (odds ratio=12, p-value=2.34E-05), 3 genes with *ischemic stroke* (odds ratio=14.4, p-value=0.0017) and 10 genes with *lung cancer* (odds ratio=14.1, p-value=2.36E-09). Network proximity of the proteins associated with these comorbid conditions to the nCoV19 host proteins may explain why patients with these conditions are increasingly affected by the viral infection. Further investigations are necessary to dissect these co-morbidities. Treatment strategies that prevent the deterioration of the underlying genetic conditions must be devised to combat COVID-19 in susceptible individuals. Additionally, neurological disorders such as *Alzheimer’s disease* (odds ratio=15.3, p-value=5.13E-07) and *schizophrenia* (odds ratio=12, p-value=4.19E-06) were also found to be enriched in the interactome, warranting further investigations into these potential co-morbidities.

### Interconnections to Ciliary Proteins

SARS coronavirus which emerged in 2002 has been known to induce necrosis in ciliated airway epithelium of humans in a species-specific manner.^55^ nCoV19’s host receptor ACE2 is highly expressed in ciliated respiratory cells.^56^ Cilia may serve as virus entry points and potential modulators of viral pathogenesis. This conjecture prompted us to investigate the ciliary association of the host proteins and their interactors in the HoP interactome. For this, we studied its overlap with an interactome of 165 ciliary proteins that we constructed in a similar manner. The ciliary protein interactome contained 1,665 proteins. 617 of these proteins, and specifically 30 core ciliary proteins, are also found in nCoV19’s host protein interactome, and the overlap was found to be statistically significant (p-value=2.24E-10, odds ratio=1.22). Thirteen novel predicted interactions connected host proteins to ciliary proteins: NUP98-CHMP5, GG3BP1-DNAH1, SEPSECS-DNAH1, NEK9-IFT43, TLE1-DNAH5, ATP6AP1-CETN2, C1orf50-ZMYND12, RAB10-IFT172, TOR1AIP1-GPR161, DNAJC19-CETN3, NLRX1-IFT46, FKBP7-TTC30B, POLA2-TMEM216 and NDUFB9-DRC7.

Pathway analysis of the 617 common proteins (i.e., common to HoP and cilia interactomes) revealed two interesting pathways: *budding and maturation of HIV virion* (p-value=1.29E-06; odds ratio=8.8) and *anti-viral mechanism by IFN-stimulated genes* (p-value=0.013; odds ratio=2.98). We predicted that ciliary protein CHMP5 involved in the former pathway interacts with host protein NUP98 which is involved in the latter pathway. This prompted us to ask whether the predicted interaction connected the functional modules of viral budding to interferon (IFN) signaling.

#### Novel interaction of NUP98 with CHMP5 may activate an IFN-stimulated pathway that interferes with viral budding

**Figure 7.**
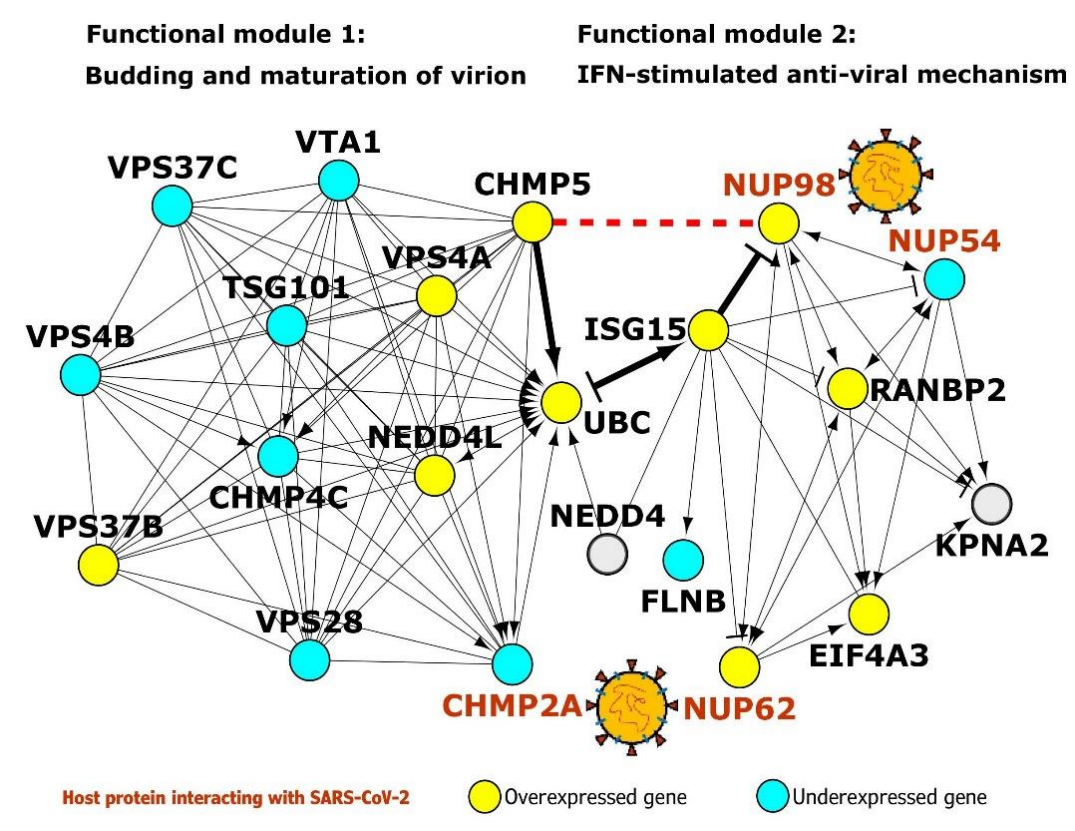
Functional modules of viral budding and interferon-mediated anti-viral pathway: The novel predicted interaction of the host protein NUP98 with CHMP5 (red dashed line) may set off an ISG15-mediated pathway that culminates in interference of viral budding. Yellow nodes and light blue colored nodes indicate overexpression and underexpression in SARS-CoV-affected Calu-3 lung cells. → indicates ‘*activation*’, -| indicates ‘*inhibition*’ and – indicates ‘*part of the same complex/physical association’*.

We extracted the PPIs of the 20 proteins belonging to viral budding and IFN pathways and isolated this sub-network, containing 171 proteins and 176 PPIs, for further analysis. Firstly, we identified 343 functional interactions (i.e. activation, inhibition etc.) among 98 proteins in the network. Strikingly, distinct functional modules were identified for both the pathways; CHMP5 seemed to serve as a connector from the viral budding pathway to the IFN pathway through NUP98 (Figure 7). The gene UBC was shared between the clusters.

We then checked whether the genes in these modules were differentially expressed in Calu-3 lung cells infected with SARS CoV Urbani (for 72 hours) versus mock infected cells. This was done to identify the functional interactions that remain active during viral infection. It was assumed that differential expression of the genes would directly impact the proteins encoded by them and their interactions. 20 genes including NUP98 and CHMP5 were found to be differentially expressed (Figure 7). Viruses hijack the ESCRT/VPS4 (*endosomal sorting complex required for transport*) machinery of the host cell to release viral particles through membrane scission.^57^ This machinery is normally recruited during endocytic and membrane repair processes in the host cell. The process of membrane scission is catalyzed by various ESCRT-III proteins including CHMP5.^57^ VPS4 is an ATPase that is found in the cytoplasm in its inactive form. Activation of the VPS4 and its ATPase activity is essential for membrane budding and the release of viral particles.^57^ VPS4 is activated on membranes in the presence of its co-activator VTA (also known as LIP5). VTA is delivered to the membranes by ESCRT-III proteins such as CHMP5.^57^ Hence, the interaction of VPS4 and VTA is facilitated by CHMP5. However, when interferons are induced in the host cell following viral infection, ISGs (*interferon stimulated genes*) such as ISG15, a dimer homologue of ubiquitin, may be activated.^57^ ISG15 may then conjugate to CHMP5 and promote its accumulation in the membrane, effectively blocking the interaction of VTA with VPS4 and preventing viral budding.^57^ The novel interaction of CHMP5 with NUP98 may serve as the critical juncture at which the IFN-stimulated anti-viral mechanism interferes with viral budding. NUP98, a protein induced on viral expression, has been shown to promote anti-viral gene expression in drosophila.^58^ Both CHMP5 and NUP98 are overexpressed following SARS CoV Urbani infection. This interaction may serve as a signal for the initiation of ISG15-mediated interference of viral budding. ISG15 may further regulate this mechanism through feedback inhibition of NUP98. Hence, potentiation of this anti-viral mechanism through administration of recombinant interferon alfa-2b and interferon alfacon-1 may be a feasible therapeutic option for nCoV19. Both these interferons induce gene expression profiles negatively correlated with SARS-associated profiles. The machinery of ESCRT-III and VPS4 is co-opted into two subcellular structures that are intricately linked to cilia function, namely, the centrosomes and the midbody.^44^ It is important to study these structures as potential modulators of viral infections.

## Discussion

In this study, to gain insight into the biological processes and pathways that may be involved in host upon nCoV19 infection, we assembled the interactome of the host proteins targeted by the virus. The host protein (HoP) interactome has ~4,000 previously known PPIs in addition to ~2,000 PPIs we computationally predicted. The interactome and its annotations are made available on the website that is freely accessible, *Wiki-Corona*. The HoP interactome was found to share a large and statistically significant overlap with a SARS-specific gene expression profile. Proteins with tissue-specific gene expression in lungs, spleen, brain and heart were also found in the interactome. Topologically connected modules in the network showed functional association to cilium organization, nuclear transport, ribonucleoprotein complex biogenesis, endosomal transport and epigenetic regulation of gene expression. The interactome is enriched for subcellular locations and host cellular processes that may be targeted by nCoV19. Host proteins were found to directly interact with proteins associated with two co-morbidities, hypertension and diabetes, that are commonly found among COVID-19 non-survivors. The nCoV19 host proteins and ciliary proteins shared several common interactors. The role of cilia as viral entry points and modulators of viral infections should be investigated further on this premise. With further analysis of the shared interactome, we hypothesize that the novel interaction of NUP98 with CHMP5, a ciliary (and centrosome and midbody-localizing) protein, may activate an IFN-stimulated pathway with the potential to interfere with viral budding.

The HoP interactome with nCoV19 associated host proteins and their interacting partners will help biologists, bioinformaticians and clinicians to piece together an integrated view on how host genes in various high throughput studies are functionally linked. To facilitate analysis by both computational and biomedical scientists, all the results are being released in multiple data formats in open access and via an interactive webserver (see Data Availability). While we carried out several analyses and presented the results here, the network of HoP interactome will facilitate several future systems biology studies derived from overlaying the interactome with data generated for research on coronaviruses in general and COVID-19 in particular. For example, consider comparing the 332 SARS-CoV-2 host proteins^6^ considered in this work, with the 65 host proteins of SARS coronavirus^59^ (‘SARS-2’ and ‘SARS-1’ respectively, for clarity). There are only 4 proteins common to them (BZW2, MARK2, MARK3 and SMOC1). However, the interactome reveals that 41 SARS-2 host proteins interact with 29 SARS-1 host proteins and that eight of these PPIs are novel PPIs (N4BP2L2-EXOSC8, NMB-MRPS5, MKRN2-MRPS25, HOXC6-BRD2, XPA-AP2M1, VKORC1- DCTPP1, RSRP1-CEP350 and TPSAB1-ADAMTS1). Gene Ontology Biological Processes such as *autophagic mechanism* (odds ratio=4.5, p-value=2.21E-05) *regulation of mitochondrion organization* (odds ratio=7.5, p-value=5.49E-05) and *protein localization to mitochondrion* (odds ratio=7.8, p-value=3.74E-04) may be commonly targeted by both these viruses as they were enriched among the proteins that are targeted by SARS-CoV-1 that also interact with host proteins of SARS-CoV-2. Mitochondria may be directly targeted by viral proteins, influenced by the cellular changes arising from viral infection or may even play a crucial role in viral pathogenesis due to their function as immune signalling hubs.^60^ These organelles are constantly eliminated and recycled through a process called mitophagy. Viruses may modulate mitochondrial function and mitophagy to exacerbate infection.^60^ Thus, in addition to the results presented here, several future studies may be carried out with the interactome to generate biologically insightful results that may be translated to biomedically actionable results.

## Methods

### Compilation of host proteins and prediction of novel interactions

Lists of 332 host proteins identified to interact with 27 nCoV19 proteins were compiled from data files in Gordon et al.^6^ Novel PPIs of these proteins were predicted using the HiPPIP model that we developed.^32^ Each host protein (say N1) was paired with each of the other human protein say, (M1, M2,…Mn), and each pair was evaluated with the HiPPIP model.^32^ The predicted interactions of each of the host proteins were extracted (namely, the pairs whose score is >0.5, a threshold which through computational evaluations and experimental validations was revealed to indicate interacting partners with high confidence). This resulted in 1941 newly discovered PPIs of the host proteins.

The significance of the overlap of this interactome with two datasets, namely, with the ciliary protein interactome and the interactome of 120 genes differentially expressed in nCoV19-infected A549 cell line,^5^ was computed based on hypergeometric distribution.

### Network functional module analysis

Network modules among host proteins and their interactors were identified using Netbox.^36^ Netbox reports modularity and a scaled modularity score, as compared with the modularity observed in 1000 random permutations of the subnetwork. Scaled modularity refers to the standard deviation difference between the observed subnetwork and the mean modularity of the random networks.^61^

### Gene expression analysis

The severe acute respiratory syndrome (SARS) dataset used was obtained from GSE17400 (Calu-3 epithelial cells infected with SARS coronavirus for 48 hours versus mock infected cells). Statistical significance of the overlap between genes in the HoP interactome and those differentially expressed in SARS was computed based on hypergeometric distribution. In this method, P-value is computed from the probability of k successes in n draws (without replacement) from a finite population of size N containing exactly k objects with an interesting feature.

$$P\left(X=k\right)=\frac{\left(\begin{array}{l} K\\ k \end{array}\right)\left(\begin{array}{c} N-K\\ n-k \end{array}\right)}{\left(\begin{array}{l} N\\ n \end{array}\right)}$$

N= Total number of genes whose expression was assayed

K= Number of genes differentially expressed in SARS

n= Number of genes in the HoP interactome

k= K ∩ n

Differential gene expression in Calu-3 lung cells infected with SARS CoV Urbani (GSE37827) and in peripheral blood mononuclear cells of SARS patients (GSE1739^40^) was checked using the Base Space Correlation Engine, a software suite for analysis of publicly available gene expression datasets.^62^ Genes with fold change >2 or <½ were considered as significantly overexpressed and underexpressed respectively at p-value<0.05.

### Tissue-specificity analysis

Tissue-specificity of the genes in the HoP interactome were checked using TissueEnrich.^63^ The analysis was based on genes from the GTEx database.^41^ This included ‘tissue-enriched genes’ with at least 5-fold higher mRNA levels in a particular tissue compared to all the other tissues, ‘group-enriched genes’ with at least 5-fold higher mRNA levels in a group of 2–7 tissues and ‘tissue-enhanced genes’ with at least 5-fold higher mRNA levels in a particular tissue compared to average levels in all tissues.

### Functional enrichment analysis

Gene Ontology (GO), Pathway and genetic disorder enrichments were computed using WebGestalt.^46^ P-values indicating significance of enrichment of the Reactome pathways, GO Biological Process and Cellular Component and OMIM diseases were corrected using the Benjamini-Hochberg test for multiple test adjustment. Annotations with FDR-corrected p-value<0.05 were considered significant. ReactomeFIViz, a Cytoscape plugin, was used to extract known functional interactions among genes in the HoP interactome.^64^ It was also used to analyse the connections between the functional modules of viral budding and interferon signalling pathways.

## Data Availability^1^

The interactome, consists of 332 host proteins, their known interactions and computationally predicted interactions. It is being released via a webserver (http://hagrid.dbmi.pitt.edu/corona) as well as in the following electronic data formats.

The list of Proteins and Protein-Protein Interactions is available in Supplementary Data File S1. The nodes and edges are labeled to indicate their type (host protein, or known or novel interactor, and known or novel PPI). The membership of the proteins in additional data sources referred to in this work can be found in Supplementary Data File S2.

## Supplementary Material

Supplement

Supplement

Supplement

## Highlights

- 1,941 novel interactions of proteins targeted by SARS-CoV-2 (‘host proteins’) are predicted with HiPPIP, our computational model.
- The interactome is made available via a web-server Wiki-Corona (http://hagrid.dbmi.pitt.edu/corona).
  - It is searchable by protein IDs and annotations.
  - More interestingly, it is searchable with queries like “Show PPIs where one protein has to do with ‘virus’ and the other protein has to do with ‘pulmonary’.”
- The interactome is made available as downloadable network files to facilitate future systems biology studies.
- Analysis of the interactome embedded with novel predicted PPIs resolved the apparent disconnect between two recent studies (transcriptional with 120 genes and proteomic with 332 genes) by showing that although they shared only two common genes, there are many direct PPIs between them and several shared common interactors.
- The interactome also showed connections between host-proteins of SARS-1 and SARS-2 coronaviruses, and the proteins common to both coronaviruses were enriched for mitochondrial proteins.
- We hypothesize that the novel interaction of the virus-induced NUP98 with CHMP5 may activate an antiviral mechanism mediated by the interferon-stimulated gene ISG15 leading to disruption of viral budding.
- The interactome had highly connected network modules which were enriched significantly for *cilium organization, cell-cell signaling by Wnt, epigenetic regulation of gene expression*, *nuclear transport*, *ribonucleoprotein complex biogenesis*, and *vesicle-mediated transport between endosomal compartments, cell cycle G2/M phase transition*, and *DNA replication*
- Midbody proteins are enriched in the HoP interactome. Viruses are known to recruit protein complexes that also localize to the midbody, a microtubule-rich stricture that marks the site of abscission during cytokinesis, to the host cell membrane, to promote scission and thereby the release of viral particles. 11 host proteins targeted by SARS-CoV-2 (RHOA, CENPF, CIT, RAB8A, NUP62, SCCPDH, SPART, RDX, ARF6, CNTRL and RALA), 63 known interactors and 9 novel interactors (KIF4A, BIRC5, INCENP, ALKHB4, DNM2, DDX11, ARL2BP, ABRAXAS2 and WIS) are midbody localized proteins.
- The interactome demonstrated significant association with 35 diseases, including hypertension, diabetes and cancers which are found to be comorbidities in critical COVID-19 cases. Additionally, enrichment for neurological disorders such as Alzheimer’s disease and schizophrenia were also found, warranting investigations into potential co-morbidities.

## Footnotes

1 Cytoscape and NDEX data will be made available at the time of publication.
