# Supplementary material for: Interactome of SARS-CoV-2 / nCoV19 modulated host proteins with computationally predicted PPIs
Source: Res Sq. 2020 May 13:rs.3.rs-28592. Preprint. [Version 1] doi: 10.21203/rs.3.rs-28592/v1 (PMC7336710; doi:10.21203/rs.3.rs-28592/v1)

Supplementary Table S1. List of experimentally validated protein-protein interactions predicted by HiPPIP. The method of experimental validation and links to source publications are also shown.

Protein 1	Protein 2	Method of validation	Publications
DDX58	OASL	Co-immunoprecipitation	https://doi.org/10.1016/j.immuni.2014.05.007
HMGB1	FLT1	Co-immunoprecipitation	https://www.nature.com/articles/npjschz201612
HMGB1	KL	Co-immunoprecipitation	https://www.nature.com/articles/npjschz201612
STT3A	RPS25	Co-immunoprecipitation	https://www.nature.com/articles/npjschz201612
STT3A	SYCP3	Co-immunoprecipitation	https://www.nature.com/articles/npjschz201612
STT3A	MCAM	Co-immunoprecipitation	https://www.nature.com/articles/npjschz201612
PDCD1	<hidden>	Co-immunoprecipitation	Unpublished validation by collaborator
ALB	KDR	In-vitro pull down and mass spectrometry	https://www.biorxiv.org/content/10.1101/459065v1 (under review at Scientific Reports)
ALB	PDGFRA	In-vitro pull down and mass spectrometry	https://www.biorxiv.org/content/10.1101/459065v1 (under review at Scientific Reports)
BAP1	PARP3	In-vitro pull down and mass spectrometry	https://www.biorxiv.org/content/10.1101/459065v1 (under review at Scientific Reports)
CLPS	CUTA	In-vitro pull down and mass spectrometry	https://www.biorxiv.org/content/10.1101/459065v1 (under review at Scientific Reports)
HMGB1	CUTA	In-vitro pull down and mass spectrometry	https://www.biorxiv.org/content/10.1101/459065v1 (under review at Scientific Reports)
STX3	LPXN	Co-localization	https://www.nature.com/articles/npjschz201612
STX4	MAPK3	Co-localization	https://www.nature.com/articles/npjschz201612
IFT88	KL	Co-localization	---
WDR5	IGFBP3	Co-localization	---

Supplementary Table S2. List of modules detected exclusively with known protein-protein interactions that are enriched for functions. Gene Ontology Biological Processes enriched in these modules are also mentioned in Column 2. Genes with * code for known interactors whereas all the other genes code for host proteins.

Genes in modules	Enriched Gene Ontology Biological Process
UBAP2L, SMURF1*, NDFIP2, PRKACA, C14orf166*, AKAP8L, UBAP2, FAM98A, STOM, RIPK1, WFS1, ASIC1*, ITPKB*, ITCH*, RHOA, RAP1GDS1, CIT, NIN, GRIPAP1, RRP9	Cell cycle G2/M phase transition<br>(p-value=0.0019, odds ratio=21.7, 20 proteins)
PABPC1, NDRG1*, CSDE1, CSNK2A2, AQP4*, GTF2F2, STC2, GPAA1, CHPF, SIX3*, GSPT2*, TCTEX1D2*, G3BP2, HDAC2, UPF1, ACSL3, AP2M1, FKBP3*, DNMT1, PPP1R8*, TLE3, CSNK2B, G3BP1, PIN1*	Cell-cell signaling by Wnt<br>(p-value=0.0049, odds ratio=9.3, 24 proteins)
POLA2, HELB*, POLA1	DNA replication<br>(p-value=0.0049, odds ratio=55.25, 3 proteins)

Supplementary Figure S1. Network view of novel protein-protein interactions: Network view of the host protein interactome is shown as a graph, where genes are shown as nodes and PPIs as edges connecting the nodes. Host proteins are shown as dark blue square-shaped nodes and novel interactors as red colored circular nodes. Red edges are the novel interactions, whereas blue edges are known interactions.

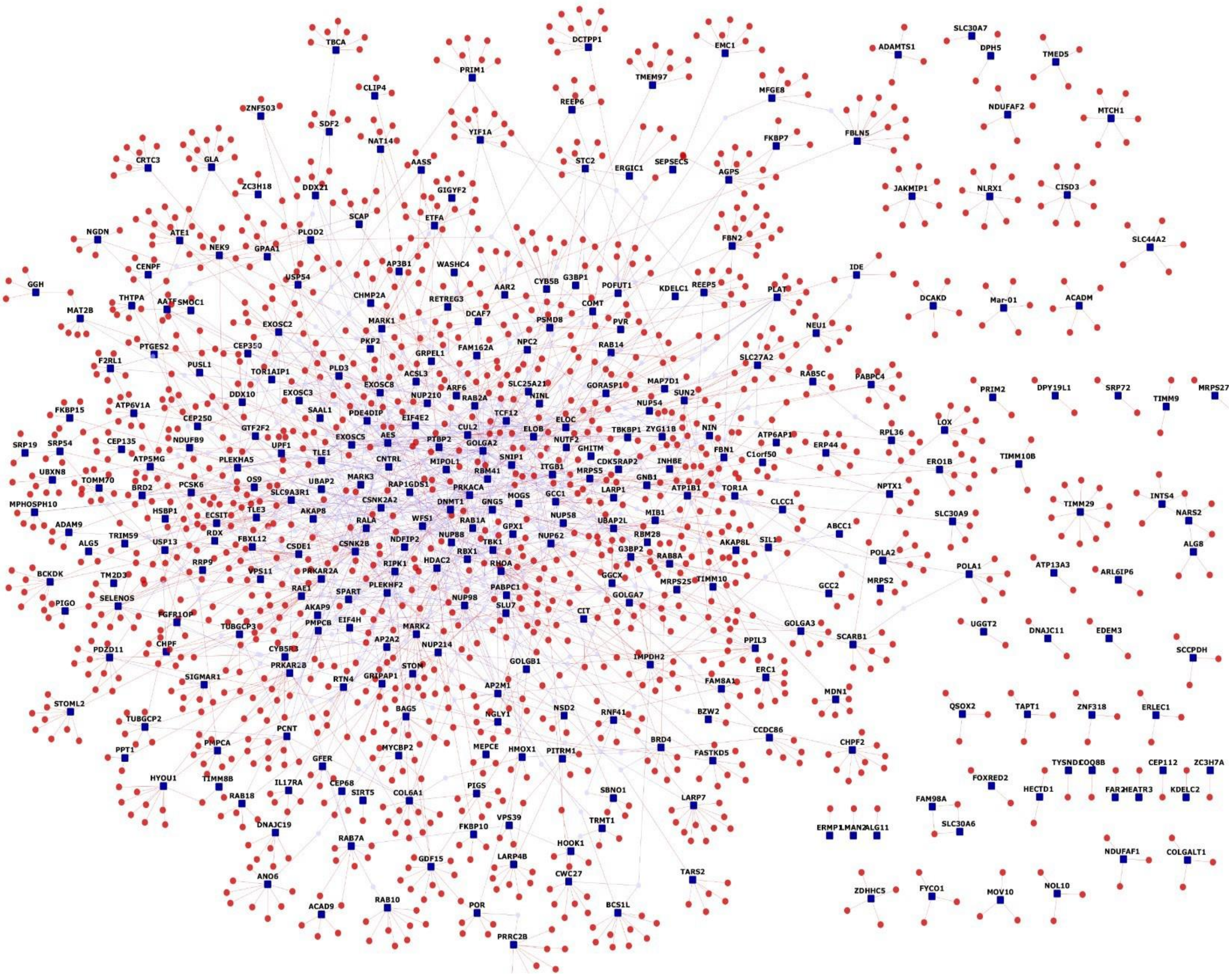

Supplementary Figure S2. Functional modules in the host protein interactome: Modules enriched in specific Gene Ontology Biological Processes are shown in a box.

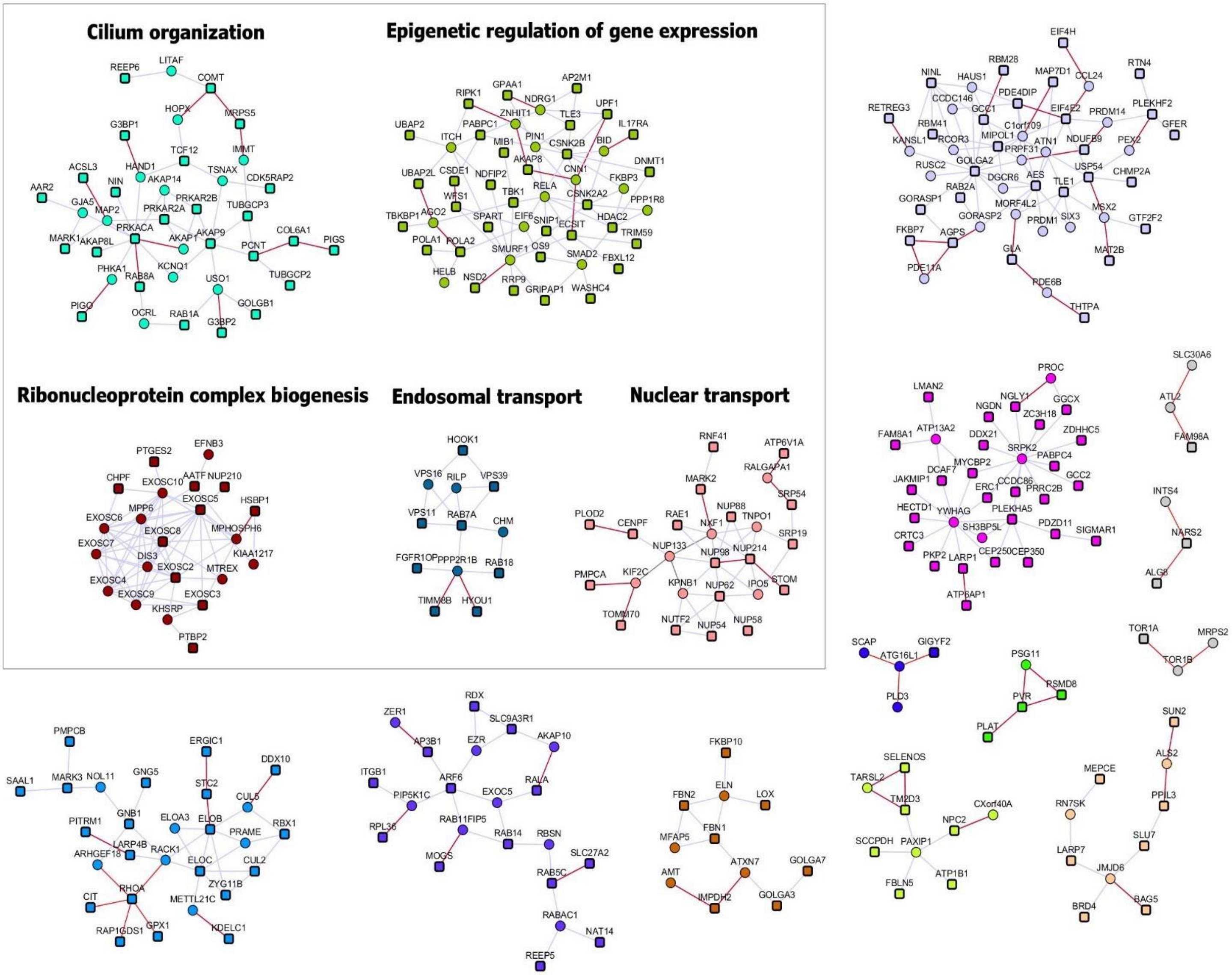

Supplement: Supplement [file SupplementaryMaterial.pdf]
